# Two-stage stochastic formulation for relief operations with multiple agencies in simultaneous disasters

**DOI:** 10.1007/s00291-023-00705-3

**Published:** 2023-01-23

**Authors:** Oscar Rodríguez-Espíndola

**Affiliations:** Aston Business School, Aston University, Birmingham, UK

**Keywords:** Humanitarian logistics, Multi-objective programming, Procurement, Simultaneous disasters, Disaster preparedness

## Abstract

The increasing damage caused by disasters is a major challenge for disaster management authorities, especially in instances where simultaneous disasters affect different geographical areas. The uncertainty and chaotic conditions caused by these situations combined with the inherent complexity of collaboration between multiple stakeholders complicates delivering support for disaster victims. Decisions related to facility location, procurement, stock prepositioning and relief distribution are essential to ensure the provision of relief for these victims. There is a need to provide analytical models that can support integrated decision-making in settings with uncertainty caused by simultaneous disasters. However, there are no formulations tackling these decisions combining multiple suppliers, multiple agencies, and simultaneous disasters. This article introduces a novel bi-objective two-stage stochastic formulation for disaster preparedness and immediate response considering the interaction of multiple stakeholders in uncertain environments caused by the occurrence of simultaneous disasters. At the first stage, decisions related to the selection of suppliers, critical facilities, agencies involved, and pre-disaster procurement are defined. Resource allocation, relief distribution and procurement of extra resources after the events are decided at the second stage. The model was tested on data from the situation caused by simultaneous hurricanes and storms in Mexico during September of 2013. The case is contrasted with instances planning for disasters independently. The results show how planning for multiple disasters can help understand the real boundaries of the disaster response system, the benefits of integrated decision-making, the impact of deploying only the agencies required, and the criticality of considering human resources in disaster planning.

## Introduction

The COVID-19 pandemic shed some light on the complexity of handling multiple co-occurring disasters. For instance, while managing the pandemic, authorities in Louisiana faced two hurricanes within 36 h, unprecedented wind speeds, and storms 2020 (Jerolleman et al. 2021). Similarly, Seattle was affected by wildfires and heatwaves (McMahon 2021). In August 2018, authorities in south Asia had to respond to floods and earthquakes at the same time (Chen et al. 2018a). The increasing number of simultaneous disasters is complicating disaster response in different countries (Trias and Cook 2021). Simultaneous disasters are “two or more disasters that temporally overlap, may be geographically distant, yet make demands of the same pool of resources” (Doan and Shaw 2019). Planning for these situations is necessary because of their cascading effects on each other (e.g., infrastructure or supply chain disruption), their varying magnitude, the different levels of vulnerability of the affected areas (FEMA 2012; Rottkemper et al. 2011), and their impact on the responses of all the stakeholders involved (FEMA 2003).

Disaster management operations involve deploying governmental agencies with diverse expertise and resources to help victims (Cozzolino 2012; Gutjahr et al. 2020). Their purpose is the dispatch of commodities to affected areas efficiently, effectively and quickly (Özdamar et al. 2004). Collaboration between local and international participants is key to manage complex environmental problems (Charles and Lauras 2011). The plurality of responders (Trias and Cook 2021), however, complicates activities because of their varying goals and access to resources (Thompson and Duintjer Tebbens 2016). Given concerns about the potential competition for resources among organizations (Altay 2013; Besiou et al. 2011), effective collaboration requires adopting a holistic view with clear coordination mechanisms to support key stakeholders (Jerolleman et al. 2021). That is the role of a central coordinator, often adopted by federal authorities (Alexander 2015), which make decisions and guide the activities of participants. Effective management of stakeholders at different levels allows to dispatch the agencies needed to support victims of simultaneous disasters (Jerolleman et al. 2021; Chen et al. 2018a), which reduces convergence of staff (Rodríguez-Espíndola et al. 2020) and mitigates the challenges with jurisdictions (Ansell et al. 2010).

Currently, the most widely used approach to handle simultaneous disasters involves adapting single-disaster mechanisms to try to make them effective in these settings (Jerolleman et al. 2021; Kappes et al. 2012). Recent experiences, however, underscore the urgency of going beyond current measures prepared for independent disasters (Chen et al. 2018a) to avoid improvising solutions once the demand caused by several disasters increases, as experienced before (Jerolleman et al. 2021). With the expected increase of simultaneous disasters in the future (FEMA 2012), disaster planners need to think about these instances to prepare effective disaster response (McMahon 2021). This research aligns to the call for having multi-hazard rather than single-hazard management systems for disasters (UNISDR 2019). Neglecting the interaction between hazards can lead to unreliable conclusions. Deciding which stakeholders to involve, what to procure and from whom to satisfy requirements at different locations simultaneously are complex issues. These decisions must consider facilities, resource constraints and demand requirements to provide for victims. Poor management in such conditions can cause delays, duplication of efforts, and inefficient use of resources, which are reasons leading governments to plan and prepare for simultaneous disasters (Doan and Shaw 2019).

This article proposes a two-stage bi-objective stochastic model to support procurement, facility location, resource allocation, and relief distribution in situations caused by simultaneous disasters as an integrated approach needed to manage humanitarian operations. The model incorporates decisions about supplier selection, facility location, stock prepositioning and organization selection at the first stage, whereas the second stage looks at resource allocation, additional procurement, and relief distribution. The formulation is tested using data from the disaster of 2013 in Mexico, where Hurricanes Ingrid, Manuel and major storms occurred at the same time and caused significant damage to the country. The results of the analysis show the importance of considering the interaction of multiple stakeholders during simultaneous disasters to prevent selecting sub-optimal solutions obtained from looking at independent disasters. The contribution is twofold: (i) it provides a novel stochastic formulation considering multiple responding agencies and multiple suppliers in humanitarian operations to manage simultaneous disasters, and (ii) it provides evidence about the impact on performance of neglecting simultaneous disasters.

The article is structured as follows. Section 2 looks at contributions combining procurement, facility location, stock prepositioning, resource allocation, and relief distribution to present the context and place the contribution in the literature. Section 3 introduces the details of the formulation, and Sect. 4 presents details of the case study. Section 5 includes the analysis of results, and Sect. 6 discusses their relevance. Finally, Sect. 7 presents the conclusions of this work and future research areas.

## Literature review

This article is looking at the combination of procurement with other logistics activities in a multi-agency environment during situations caused by simultaneous disasters. Recently, there has been a surge of articles looking at procurement to account for the impact of supply chain actors in relief operations. To understand the contribution of this article in the current state of knowledge, the literature review will look at articles focused on procurement, the evolution toward the integration of multiple participants, and at the inclusion of simultaneous disasters. Finally, it will elaborate on the research gap.

### Disaster procurement

Disaster procurement models include decisions about the suppliers required, pre-disaster products to acquire (often prepositioned), and/or post-disaster products bought and delivered. Falasca and Zobel (2011) develop a model with recourse to support procurement in disaster operations. The formulation determines how much to order right after a disaster occurs in the first stage. Once more accurate information about the impact of the disaster is received, additional quantities are requested at the second stage. The model minimizes procurement and shortage costs across all possible scenarios. Considering a risk-averse decision maker, Lu et al. (2016) provide an estimation component and a relief distribution model minimizing total travel time using a rolling horizon approach. Proposing a combination of partial prepositioning and procurement to reduce shortages of relief items, Pradhananga et al. (2016) introduce a scenario-based model minimizing a nonlinear function of social cost for supply facility selection, based on the combination of logistics and deprivation costs. Hu et al. (2017) use a scenario-based approach in a two-stage stochastic programming model to determine the number of suppliers, pre-disaster inventory levels, and locations in the first stage, while post-disaster procurement quantities and the distribution plan are determined in the second stage. The formulation minimizes cost including shortage cost and salvage value. Gonçalves and Castañeda (2018) argue that relief prepositioning decisions resemble newsvendor decisions. They use a newsvendor model and provide variants with the aim of minimizing cost. Using behavioral experiments on 20 relief managers, they show how criticality moderates demand chasing. Alem et al. (2016) design a dynamic model to minimize the weighted sum of cost and unmet demand. First-stage variables include prepositioning and vehicle contracting, while the second stage looks at relief distribution and inventory management. Hu and Dong (2019) propose a two-stage formulation minimizing total expected cost, including shortage penalties. First-stage decisions include location and pre-disaster procurement, whereas second-stage decisions involve transportation and post-disaster procurement. Alem et al. (2021) adopt a social vulnerability index to prioritize victims and needs for procurement, facility location, prepositioning, and distribution. The aim of the model is to maximize coverage, and the dynamic nature of disaster management is included using macro-time and micro-time periods. This article highlights the importance of considering the different levels of vulnerability of each community.

Articles in this section show the importance of linking pre-disaster and post-disaster procurement with other logistics activities such as relief distribution and facility location. The connection between these activities is important because of the availability of supply or supply chain constraints affecting the performance of humanitarian operations. Looking at the different formulations, the value of two-stage formulations to tackle these problems becomes evident. Nevertheless, these formulations are often designed to provide support for one organization facing a single disaster. Neglecting the interaction between stakeholders can have a steep effect on the support given to affected communities (Haimes 2012), especially considering the importance of human resources in disaster management (Santos et al. 2014). Limited resource supply, competition for resources among agencies, and poor government support (Chang et al. 2011) are problems that can be tackled considering multiple agencies and multiple suppliers.

### Disaster procurement with multiple participants

Considering the preference of governments to avoid a dependence on single suppliers for reasons of cost, image, and supply failure risk (Balcik and Ak 2014), different types of agreements looking at price and quantity for supplier selection have been studied. These studies are looking at vertical coordination, where the importance of benefits for the organization and suppliers are considered. Shokr and Torabi (Shokr and Torabi 2017) propose a coordinating platform for humanitarian organizations with two phases: bid-construction and a bid-evaluation using possibilistic models. The bid-construction model maximizes profit, whereas the bid-evaluation model minimizes cost and maximum delivery times. The use of different types of contracts has been the focus of different contributions, with articles looking at quantity flexibility, option and bonus contracts. Nikkhoo et al. (2018) analyze the use of quantity flexibility contracts to coordinate procurement in a supply chain. Their objective is to minimize cost by signing agreements between one supplier and one relief organization to coordinate the supply chain. Torabi et al. (2018) propose a fuzzy-stochastic programming model for prepositioning and procurement using quantity flexibility contracts as well. The objective of the formulation is to minimize cost, including penalty cost for unmet demand. First-stage decisions are focused on facility location and prepositioning, whereas second-stage decisions provide the post-disaster procurement and distribution plan. The SIR epidemic model is used for analysis of the post-disaster situation considering the delivery of vaccines. Shamsi et al. (2018) propose a game formulation for vaccine procurement exploring option contracts considering a backup supplier. The aim of the buyer is to minimize cost (including social cost) and the objective of the suppliers is to maximize profit. Also using option contracts, Liu et al. (2019) address governmental stock prepositioning with multiple suppliers. They formulate their model as a Stackelberg game with the government as leader and the suppliers as followers. The objective of the former is to minimize negative impact and cost whereas the aim of the latter is to maximize profit. Aghajani et al. (2020) integrate a two-period option contract with supplier selection and inventory prepositioning into a two-stage possibilistic stochastic model. The formulation has two objectives: to minimize cost and maximize coverage. At the first stage, the model determines decisions about stock prepositioning and procurement. Second-stage decisions include exercised amounts based on the contracts. Wang et al. (2019) aim to optimize agreements with suppliers for stock prepositioning. They add bonus contracts to fixed framework agreements to incentivize suppliers to reduce delivery time. They propose a performance measurement model using deprivation cost and their results show the potential of bonus contracts to improve the performance of humanitarian operations. These articles contribute to understanding the interaction between suppliers and buyers in the supply chain, but the horizontal coordination between organizations responding in the field is missing. Game theory considers different players with individual goals, which aim to maximize their own goals (non-cooperative games) or to create strategic alliances to maximize pay-offs (cooperative games) (Muggy and Jessica 2014). Horizontal collaboration has been addressed using game theory to account for competition among humanitarian organizations using non-cooperative games. Toyasaki et al. (2017) develop a newsvendor model with the aim of minimizing cost in the context of horizontal collaboration between organizations for inventory management. The joint participation of distinct NGOs is addressed by Nagurney et al. (2019) through a model capturing the competition among NGOs that determines the quantity of supplies to purchase, supplier selection, and relief distribution using specific freight logistics providers. This stream of research considers competition among players (Seaberg et al. 2017). However, when multiple players are considered, the problem becomes more difficult to solve, the participants need to be known in advance, and it is important to have foresight of information and potential strategies (Jacob and Charles 1999).

An alternative to game formulations has been to consider an upper-level coordinator directing different agencies. This approach allows to have organizations with aligned objectives, which has been addressed using resource allocation models to manage multiple participants in humanitarian logistics (Lei et al. 2015; Shan and Zhuang 2013). Celik et al. (2016) propose a formulation focused on location-allocation considering pre-disaster procurement decisions on the first stage, and post-disaster procurement and allocation on the second stage. The model aims to minimize cost considering the participation of different governmental and non-governmental organizations. Velasquez et al. (2019) account for the multi-agency collaboration between governmental agencies and other organizations for procurement, allocation, and inventory. The formulation uses robust optimization to minimize the total demand-weighted distance from distribution centers to dispensing locations. Balcik et al. (2019) introduce an insurance-based method to support facility location and stock prepositioning as part of horizontal collaboration. The model includes decisions about location, premiums, and budgets in the first stage, whereas the second stage is focused on transportation and procurement. The aim is to minimize costs and deviation from the premium paid, and the cost expected for the participants. Arif et al. (2020) focus on repairs after disasters, considering uncertainty in demand and repair times, aiming to minimize cost including penalty costs for delays. The first stage of the model determines the facilities used and the initial allocation of crews and equipment, while second-stage decisions are focused on allocating crews to affected areas and ordering new equipment.

The cluster of articles presented in this part is looking at the importance of guiding different participants in the field to improve horizontal coordination and support disaster victims. It is noteworthy that the articles in this section include resource allocation decisions as part of their formulations, given the importance of these decisions to guide activities from different participants. However, these models also neglect the potential characteristics of different disasters and its effect on the availability and allocation of resources.

### Multiple disasters and disaster procurement

Governmental authorities struggle to manage the impact and needs caused by more two or more hazards at the same time, i.e., multiple disasters. Several countries are vulnerable to face multiple hazards simultaneously (Julca 2012), and they require models to support decision-making in those conditions. Considering the expectation about the increase of multiple disasters in the future (FEMA 2012), and the reliance on shared resources to satisfy the needs of all the victims (Kappes et al. 2012), this section analyzes contributions in the procurement literature in that context.

Articles in the literature have identified different types of multiple disasters. When different hazards occur in the same community at the same time, these are called compound disasters (Wachira 1997). Klibi et al. (2018) provide a two-stage stochastic formulation to design a relief network for facility location and stock prepositioning considering the inter-arrival time between two hazards. First-stage decisions are focused on location and prepositioning activities, while second-stage decisions support relief distribution. The authors address the complexity of the need for more than one objective using a weighted sum to maximize coverage and minimize cost. Some authors have looked at situations caused by more than one disaster on different communities. Sharifyazdi et al. (2018) analyze the potential of having relief prepositioned onshore and offshore at on-board vessels. The stochastic formulation aims to support inventory and relief distribution decisions to minimize cost. The proposed model recognizes the different communities affected by hazards to determine the dispatch of products. Chen et al. (2018b) use a prepositioning policy to prepare for multiple disasters. They propose an NGO prepositioning model using the multi-product newsvendor approach to tackle multiple disasters. The flexible structure policy allows joint prepositioning among organizations to minimize cost.

Secondary disasters are another type of multiple disasters. The term refers to a series of disasters taking place after the occurrence of a primary disaster (Zhang et al. 2012). For instance, a tsunami taking place after an earthquake. Focused on those situations, Zhang et al. (2012) consider the probability of occurrence of secondary disasters as a result of the primary catastrophe to propose a resource assignment method for disaster relief. The purpose is to respond to potential secondary disasters based on the decisions made for the primary disaster. The model minimizes travel time between areas. Considering the impact of secondary or subsequent disasters in demand and delivery time, Nezhadroshan et al. (2021) design a possibilistic-stochastic model for procurement and facility location with the objective to minimize logistics costs, maximum travel time, and maximize the level of resilience of the facilities selected. Similarly, Foroughi et al. (2022) incorporate the effect of subsequent disasters on demand in a bi-objective formulation minimizing total cost and maximizing the resilience level of facilities. They show a comparison of solution methods using NSGA-II, NRGA and MOPSO.

The case where different disasters affect different areas at the same time is referred in this paper as simultaneous disasters, and it has been addressed by Doan and Shaw (2019). The authors focus on resource allocation using three optimization models: the first looking at the risk of not reaching the desired level of service with resource constraints, the second one suggesting the resources needed to satisfy the emergencies and the third one combining both models. The formulation introduces the needs of the affected areas based on the type of disaster to guide the allocation. The article shows the importance of considering available resources to carry out humanitarian operations in instances caused by simultaneous disasters.

In general, the articles in this part acknowledge the occurrence of more than one disaster in the same community and different communities. It is possible to note the importance of the difference between the areas affected and the varying nature/magnitude of the disasters. However, only Doan and Shaw (2019) consider the possibility of having independent hazards affecting different regions, which can have an effect on different logistics decisions, and it is focused on resource allocation more than on other key logistics decisions.

### Research gap

Tables 1 and 2 provide a summary of the articles reviewed. There are several key takeaways from the literature review. The possibility of congestion because of convergence of supply (Wachtendorf and Kendra 2004) has started the discussion about the value of looking at the participants and their resources. Rodríguez-Espíndola et al. (2018a) argue that selecting and sending the *required* human and material resources to the field can provide advantages over deploying all available resources. Abualkhair et al. (2020) provide evidence suggesting that having more resources does not necessarily improve the level of service. Nevertheless, most of the formulations in supplier selection and procurement found in the literature neglect to consider the interaction of multiple agencies. Game theoretical formulations make an attempt by incorporating competition between players with selfish objectives, but the introduction of stochastic parameters and the need for a set number of players complicates looking into congestion in supply. On the other hand, formulations looking at collaboration between organizations do not consider the potential impact of having an excess of participants. Hence, new formulations that can manage the resources from different participants to optimize the number of organizations involved in humanitarian operations are needed.

**Table 1:** Summary of the decisions tackled by the models

Article	Pre-disaster procurement	Post-disaster procurement	Supplier selection	Distribution	Resource allocation	Location	Type of facilities
Falasca and Zobel (2011)	✓	✓	✓				
Zhang et al. (2012)					✓		
Lu et al. (2016)		✓		✓			
Pradhananga et al. (2016)	✓	✓		✓		✓	Supply points
Alem et al. (2016)	✓	✓		✓			
Hu et al. (2017)	✓	✓	✓	✓		✓	Distribution centers
Celik et al. (2016)	✓	✓			✓	✓	Distribution centers
Toyasaki et al. (2017)		✓					
Shokr and Torabi (2017)		✓	✓	✓			
Nikkhoo et al. (2018)	✓	✓		✓			
Torabi et al. (2018)	✓	✓	✓	✓		✓	Central warehouses and local warehouses
Sharifyazdi et al. (2018)	✓	✓		✓			
Klibi et al. (2018)	✓			✓		✓	Distribution centers
Gonçalves and Castañeda (2018)	✓						
Chen et al. (2018)	✓						
Shamsi et al. (2018)	✓						
Nagurney et al. (2019)		✓	✓	✓			
Wang et al. (2019)	✓	✓					
Balcik et al. (2019)	✓	✓		✓	✓	✓	Warehouses
Velasquez et al. (2019)	✓	✓			✓	✓	Distribution centers
Liu et al. (2019)	✓						
Hu and Dong (2019)	✓	✓	✓	✓		✓	Distribution centers
Doan and Shaw (2019)					✓		
Arif et al. (2020)	✓			✓	✓	✓	Depots
Aghajani et al. (2020)	✓	✓	✓				
Nezhadroshan et al. (2021)		✓	✓	✓		✓	Central warehouses and local distribution centers
Alem et al. (2021)	✓	✓		✓		✓	Warehouses and relief centers
Foroughi et al. (2022)		✓	✓	✓		✓	Central warehouses and local distribution centers
This article	✓	✓	✓	✓	✓	✓	Distribution centers

**Table 2:** Summary of the characteristics of the models

Article	Characteristics of the models							Objectives	
	Vertical coordination	Horizontal coordination	Multiple disasters	Multiple suppliers	Multiple products	Human Resources	Stochastic demand	Multiple objectives	Objective function
Falasca and Zobel (2011)				✓	✓		✓		Minimize cost
Zhang et al. (2012)			✓		✓	✓			Minimize cost
Lu et al. (2016)				✓	✓		✓		Minimize total distribution time
Pradhananga et al. (2016)							✓		Minimize cost
Alem et al. (2016)					✓		✓		Minimize cost
Hu et al. (2017)				✓					Minimize cost
Celik et al. (2016)		✓			✓		✓		Minimize cost
Toyasaki et al. (2017)		✓					✓		Minimize cost
Shokr and Torabi (2017)	✓			✓	✓			✓	Maximize supplier profit, minimize cost efficiency and delivery time for the organization
Nikkhoo et al. (2018)	✓						✓		Maximize supplier profit, minimize cost
Torabi et al. (2018)	✓			✓	✓		✓		Minimize cost
Sharifyazdi et al. (2018)			✓		✓				Minimize cost
Klibi et al. (2018)			✓	✓	✓		✓	✓	Weighted sum of maximizing coverage and minimizing cost
Gonçalves and Castañeda (2018)									Minimize cost
Chen et al. (2018)			✓		✓		✓		Minimize cost
Shamsi et al. (2018)	✓			✓					Minimize buyer logistics and social cost, maximize supplier’s profit
Nagurney et al. (2019)		✓		✓					Maximize utility
Wang et al. (2019)	✓			✓					Minimize deprivation
Balcik et al. (2019)		✓					✓		Minimize costs and deviation from the premium paid
Velasquez et al. (2019)		✓		✓	✓		✓		Minimize demand-weighted distance
Liu et al. (2019)	✓			✓			✓		Minimize negative impact and cost for the government and maximize supplier profit
Hu and Dong (2019)				✓			✓		Minimize cost
Doan and Shaw (2019)			✓						Minimize the risk of not satisfying all resource requirements, minimize cost of additional resources
Arif et al. (2020)		✓			✓	✓	✓		Minimize cost
Aghajani et al. (2020)	✓			✓	✓			✓	Minimize cost and maximize coverage
Nezhadroshan et al. (2021)			✓	✓	✓		✓	✓	Minimize cost, maximum travel time, and resilience of the facilities
Alem et al. (2021)					✓				Maximize coverage
Foroughi et al. (2022)			✓	✓	✓		✓	✓	Minimize cost and maximize resilience of the facilities
*This article*		✓	✓	✓	✓	✓	✓	✓	Minimize cost and minimize shortage of relief

Although some models have addressed collaboration in logistics activities for single disasters, when multiple or simultaneous disasters are considered, there is an absence of articles accounting for multiple participants as well. The literature has focused heavily on compound and secondary disasters. Following the impact of climate change, the large number of natural disasters is expected to increase globally (EM-DAT 2019), which in turn will also increase the occurrence of independent hazards affecting different communities (FEMA 2012). These disasters have the added complexity of having different regions affected and lacking sequential occurrence, as opposed to compound and secondary disasters, respectively. Only Doan and Shaw (2019) have looked at simultaneous disasters in humanitarian operations, but they are focused on resource allocation alone. Their work opens the door to developing comprehensive logistics plans to support victims affected by situations caused by simultaneous disasters.

Humanitarian logistics require balancing efficiency and effectiveness (Beamon and Balcik 2008), but only Klibi et al. (2018) introduce multiple objectives in a formulation handling instances caused by multiple disasters. It is important to combine the perspective of the decision-makers and the victims to develop suitable and implementable logistics plans. Although their article does not include human resources, congestion in supply or multiple stakeholders, it shows the importance of including more than one performance measure in decision-making, which is currently understudied in the area.

Overall, the literature review has shown the lack of articles optimizing the number of participants for integrated humanitarian operations including procurement, facility location, and relief distribution, especially in instances caused by simultaneous disasters. The occurrence of simultaneous disasters and their impact on logistics activities is currently understudied. Finally, most of the formulations analyzed consider single performance measures, even though humanitarian operations need to incorporate different dimensions. This article is filling those gaps. It is the first article integrating multiple suppliers, multiple agencies, and simultaneous disasters in the same formulation. Each one of these aspects has been studied independently, but their interaction remains understudied. This article proposes a bi-objective formulation to manage logistics activities involving human resources in settings with stochastic demand and optimizing the number of agencies involved to reduce supply congestion, an approach never undertaken before in the literature.

## Methodology

### Context of the situation

The interaction of different stakeholders can have a major impact in humanitarian operations, as there is potential for duplication of efforts and competition for scarce resources (Balcik et al. 2010). Coordination plays a crucial role to facilitate the participation of multiple stakeholders and mitigate those problems. Research has shown that clear roles are essential to facilitate coordination and collaboration (Jensen and Hertz 2016). Following arguments about the value of having a coordinator overseeing and guiding operations (Akhtar et al. 2012; Takeda and Helms 2006) mention the existence of “umbrella” organizations responsible for coordinating relief activities of participants in the field. This idea is aligned with civil protection systems in different countries (Alexander 2015), where one coordinator facilitates the interaction between participants and orchestrates the response. Hence, the formulation proposed is built considering a centralized decision-making structure with an upper-level decision-maker guiding different agencies. The coordinator interacts with suppliers, manages the flow of information, deploys organizations, and allocates resources from participants to different disasters and activities.

The two-stage formulation has a clear line between pre-disaster and post-disaster activities. Prior to the disasters, it is important to set-up the relief network. That includes selecting key suppliers, which organizations to deploy, the facilities to use and pre-disaster procurement. After the disasters take place, the allocation of employees, post-disaster procurement, and relief distribution decisions are carried out. A planning horizon of 3 days before and after the disasters is considered for design, although pre-disaster activities can be implemented at any point before the expected occurrence of the disasters and the number of days for response can be tailored to the characteristics of the response system. The focus on the first 3 days after the disasters is linked to the importance of the first 72 h to provide immediate support to save lives and reduce suffering. After this period, there is a shift in needs, priorities, and resources resulting from funding becoming less available (Gustavsson 2003), aiming to provide efficient support in the medium to long term (Wassenhove 2006). As the resources from the civil protection system will be under pressure at this point, the model supports decisions to facilitate the use of resources and respond swiftly and effectively. For simultaneous disasters, once the critical first 72 h pass, the decisions from the model can be the basis for long-term dynamic formulations, whereas in cases with subsequent disasters, the information can be updated with the new information to use the model to provide support for these instances as well.

Suppliers ship relief aid to regional distribution centers, which in turn send it to the affected areas, as used by previous papers (Pradhananga et al. 2016; Hu et al. 2017). Governmental authorities often have a list of approved suppliers which provide commodities in certain amounts and with already agreed prices. Procurement of relief items can be undertaken prior to the disaster (i.e., for stock prepositioning) (Elçi and Noyan 2018; Noyan and Kahvecioğlu 2018) and after the disaster to manage the initial stages of the response based on the circumstances of the disaster.

Activities in distribution centers, such as sorting, classifying, processing and preparing for delivery (Holguín-Veras et al. 2012), are undertaken by personnel from the agencies involved. They also manage the delivery of relief using available vehicles. Similar to the case of human resources, the number of vehicles is limited and it affects the cost of operations (Briskorn et al. 2020).

The decisions described need to be made for every region affected by a disaster, and traditionally this process has been treated as independent planning in the literature. In disaster-prone countries, however, it is possible to have more than one disaster affecting different regions of a country at the same time (Doan and Shaw 2019). Therefore, this paper is proposing a stochastic bi-objective model to provide support for procurement, facility location, resource allocation, and relief distribution for multiple agencies working on situations caused by two or more disasters occurring at different regions during a similar period of time. The model can be used for planning to analyze highly vulnerable areas, or for instances in which forecasts (e.g., for hurricanes) show potential impact in different regions.

### Model assumptions

The optimization model proposed is underpinned by the following set of main assumptions:

- There is an umbrella organization coordinating disaster management participants. Having a central coordinator can help reduce duplication of efforts and competition for resources (Takeda and Helms 2006) and it has been used in previous formulations (Rodríguez-Espíndola et al. 2018b) because it aligns to the characteristics of civil protection systems in different countries (Alexander 2015).
- Shipments from the supplier to the distribution centers are arranged and managed by the supplier. This can be arranged with the supplier based on the agreements that can be established prior to the occurrence of disasters (Balcik and Ak 2014; Torabi et al. 2018).
- The agencies managing the disasters have the authority to participate in the different regions. This is an important consideration to reduce the impact of jurisdictional barriers that could affect the response to disasters occurring at different locations, which can be considered for national level emergencies (Boin and Lagadec 2000).
- Public donations are collected and managed for post-disaster distribution. Hence, these are beyond the planning horizon of the model. As the model is focused on immediate response, it is assumed that immediate response is handled with resources from official participants (Torabi et al. 2018).
- Information and resources from every participating agency and suppliers are shared with the disaster management coordinator. This assumption enables the coordinator to define the best use of all the resources available and guide effectively the response (Lu et al. 2016; Rodríguez-Espíndola et al. 2018b).
- Contracts and conditions of supply with potential suppliers are pre-arranged. This assumption is based on the type of agreement with suppliers to give more realism to the requirements for the commitment and participation of them (Balcik and Ak 2014; Torabi et al. 2018).
- Each supplier and agency involved have ready-to-ship resources and they are ready to fully contribute their resources immediately. This assumption is based on the planning horizon. The three days before the disasters strike are assumed to be enough to prepare and deploy resources based on agreements with suppliers and the interaction with the organizations involved (Balcik and Ak 2014).
- Supply capacity and costs are assumed to be known in advance. Based on the experience in previous disasters, the planning horizon of 72 h, and the use of available stock, supplier agreements are assumed to have accurate information about capacities and costs.

These assumptions shape the characteristics of the formulation. The use of a central coordinator with a clear role and attributions is essential for the formulation proposed. Centralized decision-making for disaster response is used in civil protection systems on several countries currently (Alexander 2015), which facilitates fitting the model in these cases. That coordinator enables the participation of organizations to prevent jurisdictional restrictions because they have the attributions to authorize the participation of the stakeholders. It is an approach that can be adapted to current systems and reduce cost (Arif et al. 2020). That also relies on accurate information sharing between participants and the coordinator. In the case of the central coordinator and the organizations involved being from the government, that can facilitate the exchange of information, but for external organizations this aspect needs to be carefully considered because it can affect the solutions. The interaction with suppliers can be governed by agreements. Pre-determined agreements with suppliers are set to define the requirements of supply, including the responsibilities for shipping relief, commitment fees, and order sizes (Balcik and Ak 2014). The planning horizon restricts the inclusion of donations, which can also be unpredictable, to provide a plan for civil protection authorities with their resources at hand. Overall, the assumptions presented in this section are aligned to current practice and can be achieved defining the attributions of the central coordinator, clear agreements with suppliers, and accurate knowledge about available resources at the pre-disaster stage.

### Model formulation

This article employs optimization to develop a plan for humanitarian logistics balancing the shortage of relief items with the operational cost of the logistics operations. Although authors in the literature have introduced objective functions specifically focused on humanitarian operations See Sheu (2014), Duhamel et al. (2016), the complexity of these operations can be better served using more than one objective. Thus, the use of multiple objectives has been deemed as an appropriate approach to consider the conflicting goals encountered in humanitarian operations (Beamon and Balcik 2008; Doerner et al. 2009). The model determines the optimal location of regional supply facilities (e.g., warehouses or distribution centers) based on their capacity, distance to the affected areas, and operational cost. The facilities are used to preposition stock and prepare the relief items for dispatch. These facilities are operated by staff from the organizations deployed to receive and manage items from a set of suppliers selected at the first stage of the model. Along with supplier selection, the model introduces pre-disaster and post-disaster procurement constrained by the capacity of supply from suppliers and the minimum order size set in the agreements with them. Pre-disaster procurement decisions are made at the first stage to facilitate immediate response, whereas post-disaster procurement allows decision-makers to define the quantity if relief to get immediately after the disaster to adjust to the conditions of the scenario.

Staff from the selected organizations is allocated to relief management or distribution. The purpose is to balance the needs to operate the system and avoid having an excess of human resources. On the other hand, relief distribution decisions are oriented toward the delivery of relief items from regional supply facilities to the affected areas. Relief distribution decisions include the service from supply to demand facilities, the quantity of items shipped, and the trips required for delivery.

The model is designed to provide support for disaster preparedness and immediate response, which are crucial for survival and to reduce suffering. Simultaneous disasters affecting multiple areas can be very demanding at that stage because resources must be shared quickly without a clear sequence of events. Hence, the model proposed is looking at that period, to enable decision-makers to modify once there is more information about the different damages.

Table 3 presents the model notation and definitions. The data description can be found in Appendix.

**Table 3:** Model notation and definitions

*Indices*	
*i*	Index of regional supply facilities
*j*	Index of demand areas
*o*	Index of available relief agencies
*k*	Index of potential suppliers
*n*	Index of relief items
*l*	Index of regions affected by disasters
*s*	Index of scenarios
*Scenario-independent parameters*	
${\alpha }_{k,n}$	Cost of relief item *n* from supplier *k* at stage the second stage
${\beta }_{k,n}$	Supply capacity of relief item *n* from supplier *k*
${\gamma }_{i}$	Cost of opening supply facility *i*
${\zeta }_{i,j,l}$	Cost of each trip from supply facility *i* to demand point *j* at region l
$\eta$	Number of potential trips per day per vehicle
$\theta$	Procurement budget
${\iota }_{o}$	Number of vehicles available per agency *o*
${\kappa }_{k}$	Cost of the partnership with supplier *k*
${\mu }_{o}$	Cost of involving agency *o*
${\nu }_{k,n}$	Minimum order size of relief item *n* allowed by supplier *k*
${\xi }_{i,k}$	Coverage from supplier *k* to facility *i*
$o$	Facility space covered per employee
$\sigma$	Weight capacity of each vehicle
${\tau }_{i}$	Volumetric capacity of the distribution center *i*
${\upsilon }_{o}$	Number of employees per agency *o*
${\varphi }_{n}$	Volume of relief item *n*
$\chi$	Number transportation staff required per trip per vehicle
${\psi }_{n}$	Weight of relief item *n*
${\Omega }_{k,n}$	Cost of buying relief item type *n* from supplier *k* at stage 1
*Scenario-dependent parameters*	
${\delta }_{j,n,l,s}$	Demand of relief item *n* in area *j* at region *l* under scenario *s*
${\pi }_{s}$	Probability of scenario *s*
${\rho }_{l,s}$	Priority of disaster area *l* under scenario *s*
*First-stage decision variables*	
${A}_{k}$	Selection of supplier *k*; 1 if the supplier is chosen, 0 otherwise
${X}_{i}$	Activation of distribution center *i*; 1 if the facility is opened, 0 otherwise
${Y}_{i,k,n}$	Number of relief items type *n* procured from supplier *k* for distribution center *i* at stage 1
${W}_{o}$	Involvement of agency *o*; 1 if the agency is deployed, 0 otherwise
*Second-stage decision variables*	
${C}_{i,n,s}$	Number of relief items n allocated to facility *i* at scenario s
${D}_{i,o,s}$	Employees from agency *o* allocated to relief management in DC *i* at scenario *s*
${E}_{i,o,s}$	Employees from agency *o* allocated for distribution in DC *i* at scenario s
${G}_{i,j,o,l,s}$	Trips from facility *i* to zone *j* at region *l* at scenario *s* of vehicles from agency *o*
${P}_{j, n,l,s}$	Demand not satisfied of relief item *n* at area *j* in region *l* at scenario *s*
${Q}_{i,j,n,l,s}$	Relief item type n sent from supply facility *i* to demand point *j* in region *l* at scenario *s*
${T}_{i,k,n,s}$	Relief item type *n* bought from supplier *k* for distribution center *i* at stage 2 at scenario *s*

The model supports the selection and management of the most suitable stakeholders (i.e., suppliers and relief agencies) to engage in simultaneous disasters, because of the importance of incorporating to introduce multiple stakeholders in the optimization of humanitarian operations (Anaya-Arenas et al. 2014). Having an umbrella organization coordinating the efforts of different agencies introduces the possibility of deploying only the necessary stakeholders to avoid supply congestion (Wenger et al. 1986; Abounacer et al. 2014) and supports the collaboration and swift response required to minimize death and suffering. This becomes even more relevant in situations caused by multiple disasters, to avoid competition for resources among jurisdictions (Altay 2013), to allow the holistic analysis of the use of resources, and to consider the characteristics of resource providers. The formulation represents the involvement of agencies and suppliers as decision variables to ensure only required participants with the most suitable resources are involved to handle the situation, while different regions are included as a set to account for multiple simultaneous disasters. This perspective aligns with the escalation processes for civil protection systems used in several countries (Takeda and Helms 2006).

Selected stakeholders are supported by facility and relief delivery decisions. A set of facilities are chosen to manage the relief procured for all areas before and after the occurrence of the disasters. Relief delivery involves the use of human and material resources from participant agencies. Effective use of resources in simultaneous disasters is very important. Parameter ${\rho }_{l,s}$ has been included in the model to reflect the urgency of an affected area to allow optimal resource allocation (Sarma et al. 2020) based on the nature of the disasters and the vulnerability of the areas affected. This parameter can be set using forecasts about the magnitude and nature of the events or combined with levels of vulnerability of the regions affected to determine the optimal split of resources. Hence, more severe disasters affecting vulnerable areas would have a higher ${\rho }_{l,s}$, forcing the model to use more resources to reduce shortage in those areas.

Because of the presence of uncertainty in disaster management, authorities require the development of plans to manage resources available with the aim of supporting victims (Chang et al. 2007). Hence, the model is formulated using a two-stage stochastic approach. The pre-disaster phase is addressed at the first-stage, and the second-stage is focused on the post-disaster phase See Behl and Dutta (2019). The first stage includes the activation of different agencies, the selection of suppliers, the opening of distribution facilities and pre-disaster procurement of relief sent to them. Second-stage variables involve scenario-dependent decisions at the response stage including the procurement of further relief items to complement the prepositioned stock, the allocation of human and material resources, and decisions about relief distribution. The deterministic equivalent problem of the model proposed is structured as follows:

1 $$\begin{aligned} \min \;{\text{COST}} & = \mathop \sum \limits_{i} X_{i} *\gamma_{i} + \mathop \sum \limits_{o} W_{o} *\mu_{o} + \mathop \sum \limits_{i} \mathop \sum \limits_{k} \mathop \sum \limits_{n} Y_{i,k,n} *\Omega_{k,n} + \mathop \sum \limits_{k} A_{k} *\kappa_{k} \\ & \quad + \mathop \sum \limits_{s} \left( {\pi_{s} *\left( {\mathop \sum \limits_{i} \mathop \sum \limits_{k} \mathop \sum \limits_{n} T_{i,k,n,s} *\alpha_{k,n} + \mathop \sum \limits_{i} \mathop \sum \limits_{j} \mathop \sum \limits_{o} \mathop \sum \limits_{l} G_{i,j,o,l,s} *\zeta_{i,j,l} } \right)} \right) \\ \end{aligned}$$

2 $$\mathrm{min\, SHORTAGE}= \sum_{j}\sum_{n}\sum_{l}\sum_{s}{\rho }_{l,s}*{\pi }_{s}*{P}_{j, n,l,s}$$

Objective function (1) minimizes cost whereas objective function (2) minimizes shortage of relief. The COST function involves the cost of activating a facility and preparing it for operation, staff cost incurred for activating an organization, the cost of entering a partnership with a supplier, procurement cost (prior to the disaster and after the occurrence of disaster) and transportation cost. The SHORTAGE function reduces the maximum shortage of relief items based on the priority of the region (decided according to the severity of damage and urgency) and the probability of the scenario. The constraints include:

3 $$\mathop \sum \limits_{o} D_{i,o,s} *o \ge \mathop \sum \limits_{n} C_{i,n,s} *\varphi_{n} \quad \forall i,s$$

4 $$A_{k} *\nu_{k,n} \le \mathop \sum \limits_{i} \left( {T_{i,k,n,s} + Y_{i,k,n} } \right) \le A_{k} *\beta_{k,n} \quad \forall k,n,s$$

Constraint (3) ensures enough employees are allocated to operate the supply facilities, whereas expression (4) makes sure that items procured from any supplier (either prior or after the disaster) respect the minimum order size agreed and their maximum supply capacity.

5 $$C_{i,n,s} = \mathop \sum \limits_{k} Y_{i,k,n} *\xi_{i,k} + \mathop \sum \limits_{k} T_{i,k,n,s} *\xi_{i,k} \quad \forall i,n,s$$

6 $$\mathop \sum \limits_{n} C_{i,n,s} *\varphi_{n} \le X_{i} *\tau_{i} \quad \forall i,s$$

Equation (5) combines the relief items available that have been procured at both stages, while constraint (6) ensures the total number of relief items available does not exceed the capacity of the distribution centers opened.

7 $$\mathop \sum \limits_{j} \mathop \sum \limits_{l} Q_{i,j,n,l,s} \le C_{i,n,s} \quad \forall i,n,s$$

8 $$\begin{aligned} & \mathop \sum \limits_{i} \mathop \sum \limits_{k} \mathop \sum \limits_{n} \mathop \sum \limits_{s} T_{i,k,n,s} *\alpha_{k,n} + \mathop \sum \limits_{i} \mathop \sum \limits_{k} \mathop \sum \limits_{n} Y_{i,k,n} *{\Omega }_{k,n} \\ & \quad + \mathop \sum \limits_{i} \mathop \sum \limits_{j} \mathop \sum \limits_{o} \mathop \sum \limits_{l} \mathop \sum \limits_{s} G_{i,j,o,l,s} *\zeta_{i,j,l} \le \theta \\ \end{aligned}$$

Expression (7) makes sure than only available relief items are shipped to the affected regions, whereas constraint (8) ensures the relief distribution budget is not exceeded, combining procurement and distribution cost.

9 $$P_{j, n,l,s} = \delta_{j,n,l,s} - \mathop \sum \limits_{i} Q_{i,j,n,l,s} \quad \forall j,n,l,s$$

10 $$\mathop \sum \limits_{o} G_{i,j,o,l,s} *\sigma \ge \mathop \sum \limits_{n} Q_{i,j,n,l,s} *\psi_{n} \quad \forall i,j,l,s$$

Equation (9) calculates the shortage of relief items per demand area and constraint (10) estimates the number of trips required for relief distribution. These trips are determined using capacity of the vehicles based on vehicles with the same weight capacity, which can have different volume capacities.

11 $$\mathop \sum \limits_{i} \mathop \sum \limits_{j} \mathop \sum \limits_{l} G_{i,j,o,l,s} \le W_{o} *\iota_{o} *\eta \quad \forall o,s$$

12 $$\mathop \sum \limits_{j} \mathop \sum \limits_{l} G_{i,j,o,l,s} *\chi \le E_{i,o,s} \quad \forall i,o,s$$

13 $$\mathop \sum \limits_{i} (D_{i,o,s} + E_{i,o,s} ) \le W_{o} *\upsilon_{o} \quad \forall o,s$$

Constraint (11) ensures that vehicle capacity of the agencies involved is not exceeded by the trips required, whereas expression (12) determines the number of employees to be allocated to distribution activities and constraint (13) ensures that the total number of employees allocated is below the capacity of the agencies involved. Declaration of integer and binary variables is presented below. The reason for the use of discrete variables is because of the nature of the decisions involving products, people, and trips, which cannot be divided. However, that influences the solution time.

$$\begin{aligned} & C_{i,n,s} ,D_{i,o,s} ,E_{i,o,s} ,G_{i,j,o,l,s} ,P_{j, n,l,s} ,Q_{i,j,n,l,s} ,T_{i,k,n,s} ,U_{n,l,s} ,Y_{i,k,n} , \in {\text{ Z}} \ge 0; \\ & \quad A_{k} ,W_{o} ,X_{i} \in \left\{ {0,1} \right\} \\ \end{aligned}$$

### Solution method

A major challenge of the formulation presented is that it has two objectives, which require multi-objective solution techniques. Given the added difficulty to anticipate the post-disaster decisions of each scenario, a scalarization technique is proposed to transform a vector problem into a family of scalar optimization problems (Huong and Yen 2014). The ε-constraint method has been selected because of its simplicity and the evidence in the literature about its value to obtain solutions (Nazemi et al. 2022). To implement the method, the SHORTAGE objective function has been selected as primary objective. Consequently, the COST objective function is turned into a constraint and the model takes the following form:

14 $$\mathrm{min\, SHORTAGE}= \sum_{j}\sum_{n}\sum_{l}\sum_{s}{\rho }_{l,s}*{\pi }_{s}*{P}_{j, n,l,s}$$

s.t. (3)–(13).

With the additional constraint:

15 $$\begin{aligned} & \mathop \sum \limits_{i} X_{i} *\gamma_{i} + \mathop \sum \limits_{o} W_{o} *\mu_{o} + \mathop \sum \limits_{i} \mathop \sum \limits_{k} \mathop \sum \limits_{n} Y_{i,k,n} *{\Omega }_{k,n} \\ & \quad + \mathop \sum \limits_{k} A_{k} *\kappa_{k} + \mathop \sum \limits_{s} \left(\pi_{s} *\left(\mathop \sum \limits_{i} \mathop \sum \limits_{k} \mathop \sum \limits_{n} T_{i,k,n,s} *\alpha_{k,n} + \mathop \sum \limits_{i} \mathop \sum \limits_{j} \mathop \sum \limits_{o} \mathop \sum \limits_{l} G_{i,j,o,l,s} *\zeta_{i,j,l} \right)\right) \le \varepsilon_{\Phi } \\ \end{aligned}$$

The single-objective problem is solved parametrically for different values of $\varepsilon$. The values of $\varepsilon$ are determined using the payoff table of both objectives, in which the minimum and maximum values of the COST objective function are used as reference to minimize (14) for Φ iterations. The results are used to obtain the Pareto front of the problem.

## Case study: Mexico

### Region of study

Hurricanes and tropical storms are a significant problem in the country, as shown by the 58 disasters caused by storms from 2000 to 2018 (EM-DAT 2019). However, situations are worse when different disasters affect the country simultaneously. Countries near subduction zones, such as Mexico, are more vulnerable to the occurrence of multiple hazards (Ordaz et al. 2019). From 2009 to 2018, nearly 50% of the disasters caused by hydrometeorological phenomena in Mexico occurred at the same time as other disasters (EM-DAT 2019). For instance, in September 2013, Hurricane Ingrid affected the Caribbean region, while Hurricane Manuel approached the Pacific coast, and heavy rainfall was reported in the northwest of the country. These disasters affected around 155,000 people and caused nearly 200 deaths (EM-DAT 2019). The case study analyzed in this article is based on that situation because of the economic and social implications of reacting to a complex situation caused by the three disasters with limited resources.

### Disaster management structure

Decision-making in disaster situations in Mexico is centralized, with the National System for Civil Protection (SINAPROC) as the coordinating entity in charge of articulating activities from different participants (Sosa-Rodríguez 2006). Each organization has regulations and directives in disaster situations (SEGOB 2006), often overlapping. The SINAPROC directs other branches of the government and collaborators to deliver the response as per government legislation (Alexander 2015). SINAPROC relies on the organization in charge of food services (DICONSA), responsible for procuring and distributing food for social programs and disaster relief, and to use their pre-arranged agreements with suppliers to source relief.

### Data collected

Data were collected from secondary sources through reports and freedom of information (FOI) requests. Freedom of information requests are submitted electronically through the Mexican transparency system including the type of request, the information required and the responsible agency. Information was gathered from eight agencies in health services (IMSS), DICONSA, relief activities (Red Cross), family services (DIF), police (SSM), military (SEDENA and SEMAR) and civil protection (Civil Protection), as well as the Mexican National Institute of Statistics and Geography (INEGI).

Information about suppliers, facilities and costs was collected from DICONSA. SINAPROC provided data about demand, relief distributed and emergency declarations. Geographical information was obtained from INEGI. Regulations from FONDEN were used to gather the characteristics of the different relief items considered. Transparency websites from the Mexican government were used to gather information about budgets and wages, whereas SEDENA provided information about the number of employees required for distribution. Each organization provided information about their employees and vehicles available, including their characteristics. The information can be classified into five categories: demand areas, procurement and relief, relief agencies, distribution network, scenario development.

#### Demand areas

The regions were obtained from emergency declarations made by the Mexican government including the period from September 16th to September 30th of 2013. The areas were clustered, based on their geographical location, into three regions shown in Table 4.

**Table 4:** Regions affected by Hurricanes Ingrid, Manuel and heavy rainfall

States (Southwest/center)	Affected people	States (East)	Affected people	States (Northwest)	Affected people
Guerrero	238,028	San Luis Potosí	46,926	Colima	15,523
Oaxaca	13,618	Nuevo León	3,663	Jalisco	31,598
Chiapas	15,746	Quintana Roo	14,263	Zacatecas	11,001
Morelos	4014	Tamaulipas	29,958	Chihuahua	60,250
Michoacán	49,368	Veracruz	7,555	Nayarit	9,762
				Sinaloa	18,497

*Source* Compiled by author with information from SEGOB

The summarized demand can be seen in Table 13 in Appendix.

#### Procurement and relief

The 39 suppliers that have participated in previous disasters were incorporated into the analysis. The cost per relief item per supplier at stage one was obtained from records of previous purchases, while the cost of purchase at stage two was assumed to be 20% more expensive. The cost of partnering with a supplier during disaster relief was assumed based on the frequency of participation in previous disasters as the information was privileged. Minimum order size was assumed to be only 1% of the supply capacity of the organization and procurement budget was obtained from reports from previous disasters (SEGOB 2008). Table 5 introduces the characteristics of the suppliers incorporated in the analysis, which include the variety of relief items offered and supplier size.

**Table 5:** Characteristics of the suppliers analyzed

ID	Products	Size	ID	Products	Size	ID	Products	Size
1	4	Large	14	1	Medium	27	1	Medium
2	1	Medium	15	1	Medium	28	1	Medium
3	1	Small	16	1	Medium	29	1	Medium
4	1	Medium	17	1	Medium	30	1	Small
5	1	Medium	18	1	Medium	31	1	Medium
6	1	Medium	19	1	Large	32	1	Large
7	2	Large	20	1	Small	33	1	Large
8	2	Small	21	1	Medium	34	1	Large
9	1	Medium	22	2	Large	35	1	Large
10	1	Medium	23	3	Large	36	1	Large
11	1	Large	24	1	Large	37	1	Large
12	1	Large	25	1	Medium	38	1	Medium
13	1	Medium	26	2	Small	39	1	Medium

*Source* Compiled by author with information from DICONSA

The relief items are the components of the food kit distributed by authorities in disasters for four people for four days (SEGOB 2012).

#### Relief agencies

Human and material resources available for disaster response were collected from FOI requests, transparency websites and reports from Red Cross. Table 6 shows the eight organizations that acknowledged participating in operational activities during the disaster, their personnel available, their wages and their vehicles available. The vehicles included were assumed to have a capacity of 2 tons.

**Table 6:** Resources of the organizations included

Organization	Staff (people)	Vehicles (truck)	Wages (MXN)	Organization	Staff (people)	Vehicles (truck)	Wages (MXN)
DICONSA	54	22	62,330	Red Cross	72	185	56,410
DIF	72	11	65,283	SEDENA	502	182	644,300
IMSS	30	0	23,504	SEMAR	1324	41	1,699,310
Civil Protection	28	4	21,937	SSM	65	0	50,926

*Source* Compiled by author with information from each organization

#### Distribution network

**Fig. 1:**
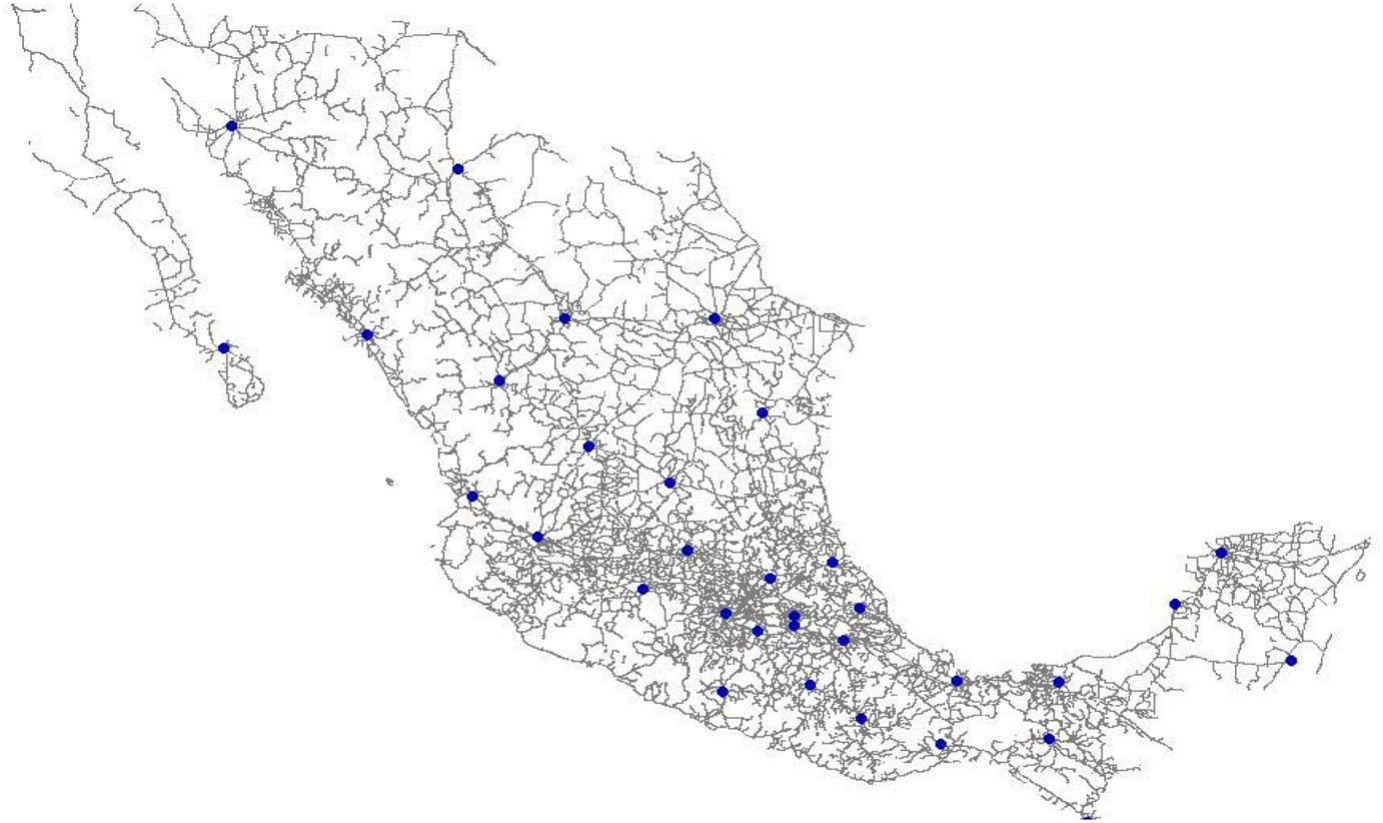
Location of potential supply facilities

The facilities used were obtained from DICONSA, geo-referenced and identified using Google Earth®, and they were located in the network of the country using TransCAD® as shown in Fig. 1.

Similarly, supplier warehouses were mapped using layers from INEGI with TransCAD®. The main supplier warehouses were used because these facilities were expected to be used to consolidate the volume of relief items required. Network analysis was performed to calculate distances between facilities, which were used to estimate transportation costs using fuel requirements.

#### Scenario development

The scenarios were based on information of the disasters affecting Mexico in September 2013. The simultaneous impact of Hurricane Ingrid, Hurricane Manuel, and heavy rainfall was reflected in the emergency declarations of Mexico from September 16th until the end of the month.

The potential scenarios were planned considering three hazards with different impact on different regions. Three levels of impact were included: regular impact (based on emergency declarations of the disasters from FONDEN (2013)), increased impact (increase of 25% over the recorded demand), and null impact (to account for the uncertainty of the hazard causing damage to the region). The probability of the scenarios was based on historical information. The variation of the impact of disaster for the development of scenarios has been used in past papers See Falasca and Zobel (2011), Balcik and Ak (2014). Using the database EM-DAT (EM-DAT 2021), a list of 149 disasters from 2000 to 2020 was included in the analysis. The list was filtered to include disasters that occurred and ended on the same months. That resulted in a total of 31 situations with 2 or more hazards affecting the country. The frequency of damage on the different regions was used to identify the probability of occurrence of disasters in each region shown in Table 2. Using the different combinations of impact on each scenario, normalizing the probabilities, and weighting them depending on the relative probability of occurrence of two (67.7%) or three simultaneous hazards (32.3%) obtained from the frequency from historical data, the final probabilities obtained are shown in Table 7.

**Table 7:** Scenarios tested

Scenario	R1	R2	R3	Probability	Scenario	R1	R2	R3	Probability
S1	I	I	I	0.005040	S11	R	I	N	0.056077
S2	I	I	R	0.015121	S12	R	R	I	0.045363
S3	I	I	N	0.018692	S13	R	R	R	0.136089
S4	I	R	I	0.015121	S14	R	R	N	0.168231
S5	I	R	R	0.045363	S15	R	N	I	0.047484
S6	I	R	N	0.056077	S16	R	N	R	0.142453
S7	I	N	I	0.015828	S17	N	I	I	0.007818
S8	I	N	R	0.047484	S18	N	I	R	0.023455
S9	R	I	I	0.015121	S19	N	R	I	0.023455
S10	R	I	R	0.045363	S20	N	R	R	0.070364

*I* Increased, *R* Regular, *N* None

## Analysis of results

### Results of the case study

**Fig. 2:**
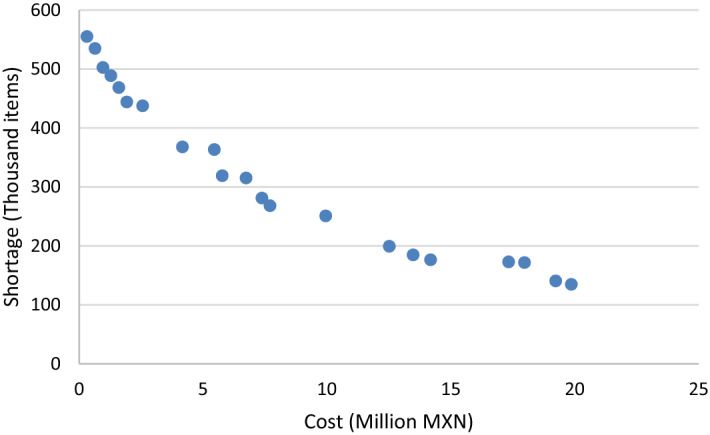
Pareto frontier of the case study

The model introduced was programmed on GAMS 23.5.1® using the ε-constraint method with the COST objective function turned into a constraint. The model was solved using Cplex® for 100 iterations. The stopping criterion was a relative optimality gap smaller than 0.1 or after 14,400 s (Kappes et al. 2012; Chang et al. 2011). The software returned a total of 21 non-dominated solutions, i.e., solutions which are not improved in both cost and shortage level by any other solution in the feasible space. The solutions can be seen in Fig. 2, which shows the trade-off between both objective functions. Each one of the non-dominated solutions includes an entire policy to manage procurement, facility location, resource allocation, and relief distribution.

Table 8 introduces a summary of the policies suggested by the optimization model. The importance of considering the resources required is noticeable in the table. As more employees are deployed for managing and delivering relief items, more items can be procured for delivery to victims. This highlights the importance of considering the interaction between human and material resources in disaster management models to augment decision-making, which is a dimension often overlooked in the literature. Another important finding is that the number of organizations deployed rely on the resources available and the level of service provided. This affects the complexity of the relief network and management of scarce resources.

**Table 8:** Summary of results of the non-dominated solutions

ID	Cost	Shortage	Supply facilities	Agencies	Suppliers	Employees used (Max)	Items procured (Max)	Trips (Max)
ND1	320,999.99	554,862	2	2	1	83	94,901	16
ND2	641,999.98	534,616	2	3	2	143	195,852	27
ND3	962,999.98	502,430	2	3	2	143	197,295	28
ND4	1,283,999.96	488,456	2	4	3	188	292,287	31
ND5	1,605,000.00	468,264	5	4	4	168	302,186	28
ND6	1,925,999.99	443,838	4	4	4	188	330,967	36
ND7	2,567,999.98	437,426	12	5	6	242	427,547	37
ND8	4,172,999.97	367,664	11	6	6	631	564,082	119
ND9	5,456,950.40	363,276	17	4	7	1268	730,225	204
ND10	5,777,999.99	318,826	16	5	8	1291	694,560	204
ND11	6,741,000.00	315,014	18	4	8	1268	712,383	201
ND12	7,382,999.94	280,943	19	4	9	1268	752,032	207
ND13	7,703,999.98	268,023	18	4	9	1268	769,556	213
ND14	9,950,999.94	250,630	22	7	11	1763	960,109	310
ND15	12,518,999.99	199,142	27	7	14	1734	1,103,904	319
ND16	13,481,999.95	184,458	24	7	17	1763	1,198,847	324
ND17	14,184,320.02	176,136	30	8	25	1788	1,107,477	325
ND18	17,333,999.98	172,548	20	5	16	1686	1,265,192	321
ND19	17,975,999.67	171,545	26	8	20	1788	1,152,713	298
ND20	19,237,919.48	140,320	31	8	28	1788	1,203,903	325
ND21	19,867,589.33	134,592	31	8	27	1788	1,335,910	325

The different combinations found in the solutions show the way the model balance different decisions. For instance, ND17 and ND18 have different balances of pre-disaster and post-disaster procurement. ND17 has the largest number of items acquired at the second stage (over 90% of relief in that solution), whereas ND18 has the largest number of items procured prior to the disaster of all the non-dominated points (914,322). The former increases procurement cost but reduces transportation cost, as opposed to ND18. Service-oriented solutions require the involvement of more agencies, facilities, and suppliers, as expected. Overall, it would be complicated to satisfy the needs of the different disasters with the resources available, as the model was unable to provide solutions without shortage.

**Fig. 3:**
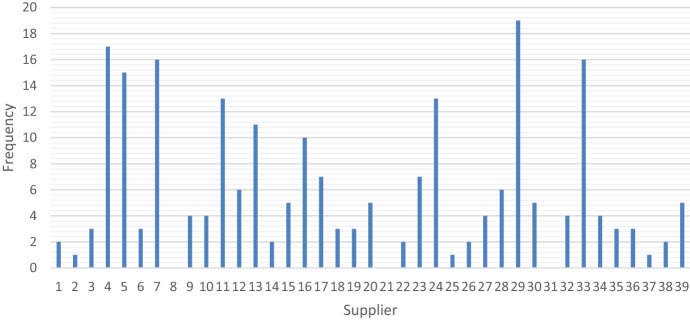
Frequency of selection of the facilities to serve as distribution centers

The model selected suppliers based on the demand, supplier characteristics and overall supply capacity. None of the solutions included all the suppliers available, showing preferences for some of them. Most solutions selected suppliers located toward the center of the country such as 4, 7, 29, and 33. The general frequency can be seen in Fig. 3.

**Fig. 4:**
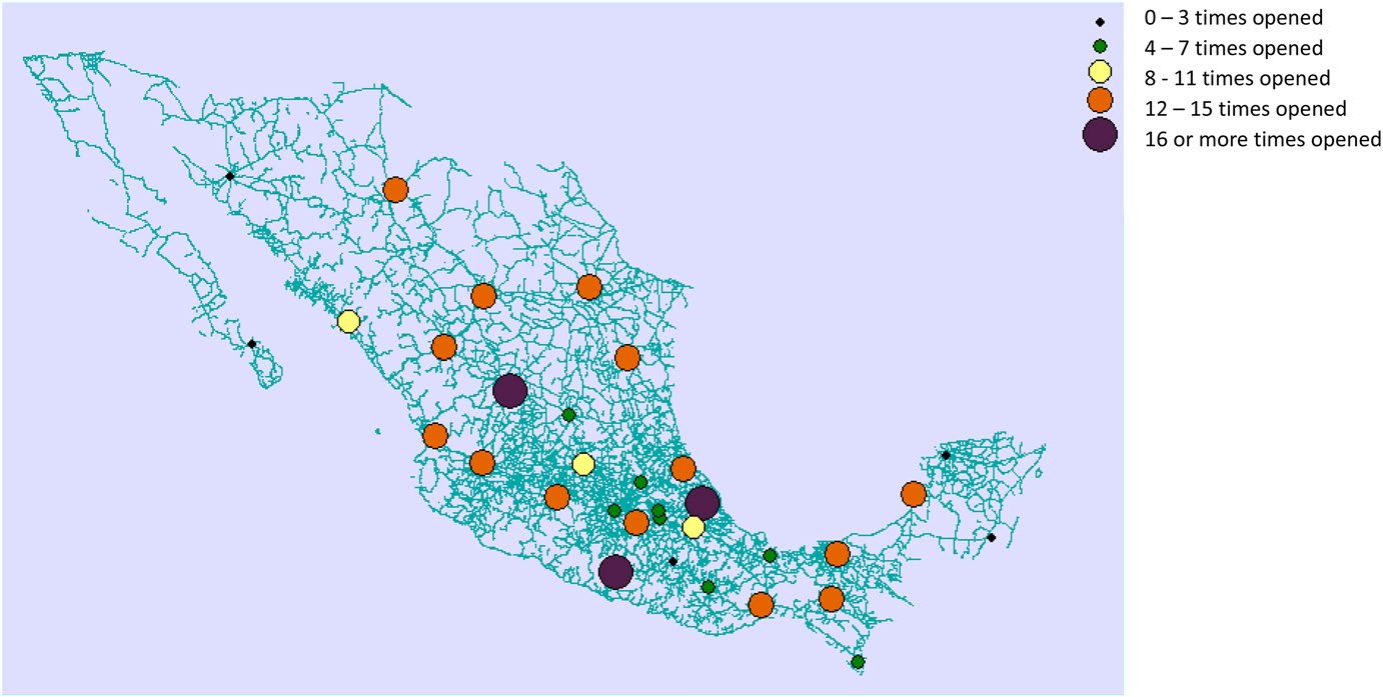
Frequency of selection of the facilities to serve as distribution centers

Figure 4 shows the opening frequency of each one of the facilities included in the analysis. Facilities in Chilpancingo, Zacatecas, and Veracruz are commonly opened in the results, with the facility in Zacatecas being opened in every single solution except for ND3. On the other hand, facilities in La Paz and Quintana Roo are never opened, whereas facilities in Mixteca and Hermosillo are opened just in a couple of the solutions. Given the distribution of the three regions affected the model supports the selection of facilities in the center of Mexico rather than facilities on the edges of the country.

**Fig. 5:**
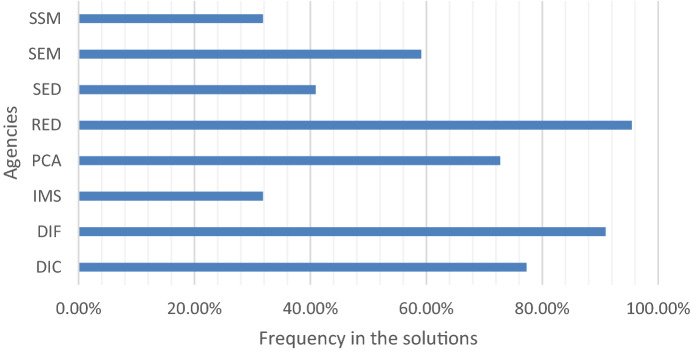
Frequency of deployment of the agencies

Figure 5 shows the percentage of activation of the agencies in the non-dominated solutions. The Red Cross, family services, DICONSA, and civil protection are activated in most of the solutions because of the combination of resources they bring to the field. Despite its cost, SEMAR is consistently selected on all the service-oriented solutions because of the large number of employees that can be allocated to relief activities.

**Fig. 6:**
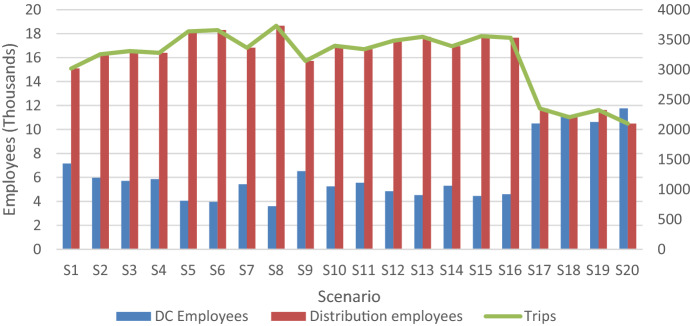
Total trips and personnel allocated across solutions

There are some interesting results in the second-stage variables as well. The restriction in the number of employees available forces the model to balance resources between distribution centers and relief distribution as shown in Fig. 6. The picture across all non-dominated solutions shows preference for using staff for relief distribution in most of the scenarios except for scenarios 17, 18, 19, and 20. Interestingly, in those scenarios, only regions two and three are affected, both of which have less demand than region one. When there is a disaster in the latter, the model focuses on using staff for distribution to facilitate reaching the affected people, whereas in the former less trips are required and more staff can be allocated to distribution centers to manage relief.

**Fig. 7:**
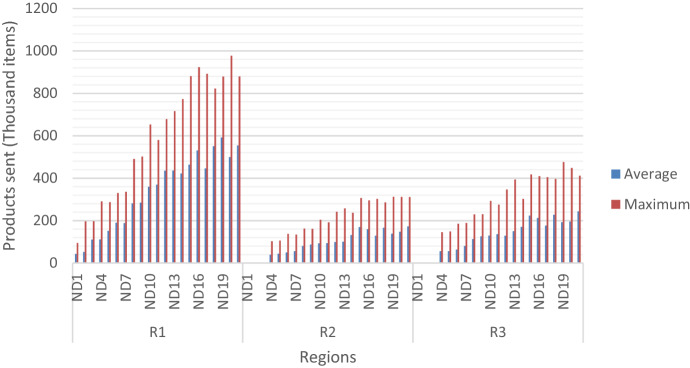
Maximum and average relief sent per solution

The magnitude of delivery to each region can be seen in Fig. 7. It shows the average and maximum number of items sent per region on every solution. Evidently, demand on region 1 represents a significant proportion of the overall demand and the need for deliveries. Additionally, the difference between the average and maximum number of items send shows the range of variation across scenarios.

**Fig. 8:**
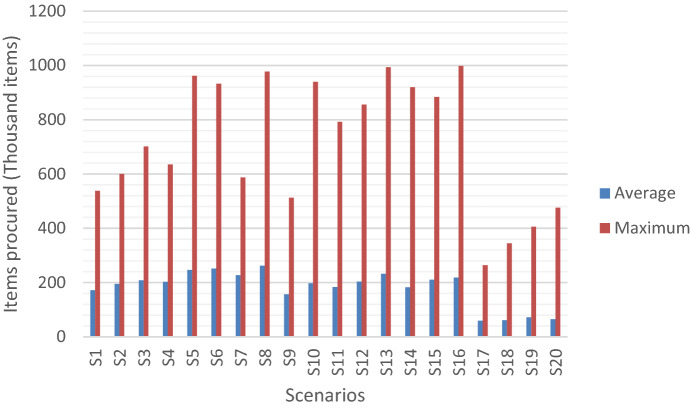
Average and maximum number of items procured per scenario

That range is reflected on the number of items procured at the second stage as well with a significant variation across scenarios as shown in Fig. 8.

### Value of the stochastic solution

The use of a two-stage stochastic approach is based on its benefits to provide reliable solutions considering the conditions of disaster management operations. This approach is commonly evaluated using deterministic approaches such as the *Expected Value of Perfect Information* (EVPI) and the *Value of the Stochastic Solution* (VSS). These measures are used to explore the performance of the results of the model using the results of the recourse problem (RP). This section presents the results of the EVPI and VSS analysis.

EVPI is based on the idea of having accurate information about the situation, which in this case involves knowledge about the surge in demand caused by the different hazards. Assuming the availability of perfect information, it is possible to solve one optimization model for each scenario. The Wait-and-See (WS) solution is obtained by aggregating the expectation of all the scenarios given by ${z}^{\mathrm{WS}}=\sum_{s\in S}{\pi }_{s}*{z}_{s}^{\mathrm{WS}}$ (Rodríguez-Espíndola et al. 2020). Hence, in this article 20 deterministic problems are solved for each epsilon value, weighted by their probability, and aggregated to obtain the WS value. EVPI is calculated with those values using the expression $\mathrm{EVPI}={z}^{\mathrm{RP}}-{z}^{\mathrm{WS}}$ (Birge 1982).

The expected value problem (EV) is obtained when the stochastic parameters are replaced by their average values of to simplify the analysis. The problem becomes easier to solve, but the probability and information from the different scenarios is neglected. To evaluate the use of the EV solution, VSS is calculated (Rodríguez-Espíndola et al. 2020). The first-stage variables are pre-fixed using the results from the EV solution, and the simplified problem is solved to obtain the expected value of using the EV solution (EEV). VSS can be calculated using the expression $\mathrm{VSS}={z}^{\mathrm{EEV}}-{z}^{\mathrm{RP}}$ (Birge 1982). Table 9 shows the summary of the results of the analysis.

**Table 9:** Analysis of the stochastic value of the solutions

Solution	RP*	WS**	EVPI	EV	EEV***	VSS
*ND1*	554,862	553,964	898	475,169	592,041	37,179
*ND2*	534,616	524,985	9631	475,169	592,041	57,425
*ND3*	502,430	499,122	3307	475,169	592,041	89,612
*ND4*	488,456	476,062	12,394	475,169	592,041	103,585
*ND5*	468,264	455,683	12,581	475,169	592,041	123,778
*ND6*	443,838	437,384	6453	475,169	592,041	148,204
*ND7*	437,426	410,651	26,775	357,117	592,041	154,615
*ND8*	367,664	324,965	42,700	338,154	592,041	224,377
*ND9*	363,276	286,612	76,664	250,113	459,517	96,241
*ND10*	318,826	269,890	48,937	239,041	445,858	127,032
*ND11*	315,014	231,158	83,856	207,427	381,993	66,980
*ND12*	280,943	213,634	67,310	194,497	352,381	71,438
*ND13*	268,023	205,404	62,619	174,431	324,949	56,927
*ND14*	250,630	145,985	104,645	118,105	242,295	–
*ND15*	199,142	113,363	85,778	96,323	199,486	345
*ND16*	184,458	104,998	79,460	85,014	182,247	–
*ND17*	176,136	59,302	116,834	56,490	98,672	–
*ND18*	172,548	77,359	95,190	66,230	134,297	–
*ND19*	171,545	73,166	98,379	62,483	133,523	–
*ND20*	140,320	59,133	81,187	56,490	98,666	–
*ND21*	134,592	59,328	75,264	53,585	82,226	–
Average	322,524	265,817	56,708	247,929	374,878	96,981
Minimum	134,592	59,133	898	53,585	82,226	345
Maximum	554,862	553,964	116,834	475,169	592,041	224,377

*Average optimality gap of 25.93%, **Average optimality gap of 0.75%, ***Average optimality gap of 19.61%

The objective was to minimize the shortage of products to reduce suffering. The EVPI values show that randomness plays an important role in the problem. The availability of perfect information would allow us to deliver over 56 thousand products in average. In the best-case scenario, perfect information would allow us to deliver 898 more items, whereas in the worst-case scenario that figure increases dramatically to 116,834. It is possible to notice that in most instances EEV values are higher that RP values. That is because using the EV solution prevents from minimizing shortage based on the value of each random variable. The results of the VSS show significant variation between the average (96,981), worst-case (224,377) and best-case scenario (345). The reason some EEV values go below the RP values is related to the optimality gap. In instances where the difference between the optimality gap of the RP problem and the optimality gap of the EEV problem was 12.4% or higher, that behavior was observed. That suggests the need to develop algorithms that can reduce the optimality gap in sensible solution times. Overall, the analysis shows that uncertainty is an important factor in the problem.

### Results of the independent analysis of simultaneous disasters

Most models in the literature consider single disasters managed by a single organization, which ignores the challenges associated with having multiple agencies involved in different events. The case study presented in Sect. 4 is used to compare the performance of the model proposed with situations considering independent decision-making for each disaster.

The underpinning assumption in several disaster management models is that there is a focal disaster requiring all the support. That assumption can tempt regional civil protection authorities to produce plans looking at single disasters. Planning for each disaster independently, without considering other events, can affect the availability of resources that need to be shared. To explore those challenges, the first experiment is assuming each one of the hazards is planned for and managed independently. In the same way, all the resources are available to support in the focal disaster when authorities assume the occurrence of a single disaster, the first experiment involved creating one model for each disaster (using the demand of that region alone) and solving it under the assumption that all the resources are available. One model per region was solved for 50 iterations under the same conditions as the original model for comparability. The results of the three models were aggregated and are presented in Table 10, which shows the solutions obtained. Looking at the maximum number of employees available per scenario, there were only six feasible solutions requiring less employees than the number currently available (i.e., 1778). The reason is because the needs of the other events are not considered, which can complicate sharing resources. In reality, that would translate in requests for resources that would be denied because of the prior depletion of resources, or the sub-optimal split of resources. The feasible solutions are dominated by solutions from the model. For instance, solution R6 (the feasible solution with the lowest level of shortage) has nearly twice as many shortage as solution ND21 from Table 8. The reason is because the results are focusing on local optimums.

**Table 10:** Details of the solutions from the aggregated planning of independent disasters

ID	Cost	Shortage	Supply facilities	Agencies	Suppliers	Max Employees available	Maximum trips
R1	1,461,000	498,327	4	2	2	226	41
R2	2,922,000	432,582	7	3	5	406	74
R3	4,379,138	398,316	11	5	7	749	93
R4	5,844,000	340,642	9	6	9	1078	188
R5	7,304,999	318,282	11	6	12	1101	174
R6	8,765,999	265,749	11	5	15	1482	271
R7	11,688,000	253,010	15	7	15	2926	299
R8	13,140,810	209,382	11	4	14	2672	376
R9	14,610,000	173,982	22	7	19	2260	404
R10	17,532,000	145,501	27	8	24	2334	427
R11	18,992,999	117,026	13	5	16	3477	462
R12	20,453,999	101,271	17	5	17	3565	462
R13	23,375,734	79,451	26	3	20	3750	521
R14	24,795,579	68,816	27	6	19	3915	569
R15	27,705,017	59,148	29	8	24	4062	552
R16	29,213,981	58,653	31	8	29	4623	669
R17	30,678,135	50,940	30	8	35	4205	577
R18	32,129,358	46,714	30	8	35	4996	825
R19	33,601,645	43,056	30	8	38	4677	600
R20	36,519,176	35,624	30	8	39	5117	835
R21	38,428,723	28,085	31	8	39	5132	820
R22	39,674,911	22,485	31	8	39	5202	951
R23	40,600,826	16,631	31	8	39	5132	948
R24	41,557,575	12,423	31	8	39	5155	950
R25	44,976,049	11,338	31	8	39	5339	959
R26	48,051,764	10,218	31	8	39	5260	971

For further analysis, it is possible to look at a combination between integrated planning and independent response. This combination would allow collaborative planning to define the relief network considering the possibility of having multiple disasters but managing the response looking at the needs of individual local authorities. This can be achieved by solving the integrated model first to fix the results of the first-stage variables and then solving one model for each region to determine the value of the second-stage variables. That way, the same relief network is used to optimize independent second-stage decisions. The results of the analysis are shown in Table 11. The findings show that as each hazard is being handled independently at the second stage, several solutions become unfeasible because of the competition for resources (i.e., employees). The reason is because this approach can generate duplication of efforts on the second stage, and it is not considering the links between regions.

**Table 11:** Solutions of the combination of integrated planning and independent response

Sol	Cost	Shortage	Supply facilities	Agencies	Suppliers	Max employees	Max products purchased	Maximum trips
ID1	0	592,042	0	0	0	0	0	0
ID2	3,433,967	455,961	7	4	3	657	272,664	69
ID3	10,329,225	378,282	17	4	4	3804	735,556	247
ID4	9,233,939	324,656	30	6	9	4041	893,540	478
ID5	12,481,346	270,992	18	4	6	3804	1,098,791	516
ID6	13,195,849	212,802	18	4	7	3804	1,187,284	553
ID7	20,907,317	174,835	30	7	15	5295	1,273,129	447
ID8	26,141,978	143,213	19	5	10	3879	1,332,622	553
ID9	31,715,539	89,802	30	7	27	5295	1,437,356	700
ID10	42,630,053	41,360	20	6	12	3948	1,571,201	490

**Fig. 9:**
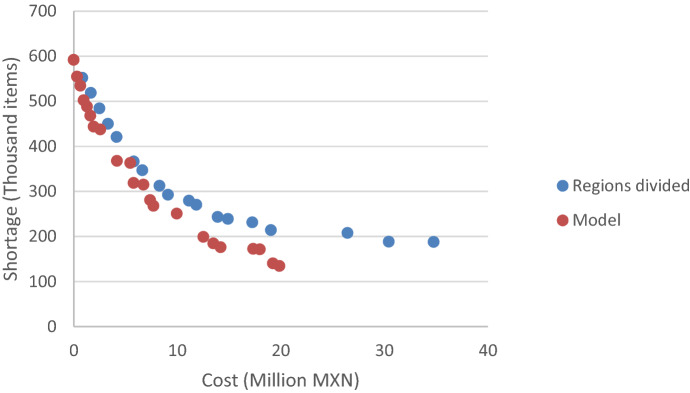
Comparison of the Pareto front between the results of the model and independent disasters

As the solutions above would be difficult to use in practice, the next test was to allocate a part of the resources a priori to each area for comparison. Each disaster was assumed to be independent, but the split of resources was made before any decisions were defined. As each disaster is considered as an independent event, one model is solved for each disaster. Assuming that each disaster was equally important for authorities, each region was allocated a third of the resources including budget, vehicles, human resources, and quantity of relief items that could be obtained from suppliers. The comparison between the Pareto front of the model and the results of splitting results a priory for independent disasters can be seen in Fig. 9. It is possible to notice that the difference between both frontiers increases in more service-oriented solutions. The use of independent relief networks implies added cost and extra use of resources for operations looking to satisfy the demand as much as possible.

Table 12 shows the comparison between the solution with lowest shortage in the model and the solution with lowest shortage from independent operations. The results show a consistent need for more complex relief networks requiring more suppliers and resources. As each one of the disasters was managed separately in the second instance, resources were allocated to satisfy local needs rather than taking advantage of pooling and sharing resources and facilities, which can provide advantages for operations (Balcik et al. 2019).

**Table 12:** Comparison of the solutions with minimum expected shortage

Solution	Cost	Shortage	Facilities	Agencies deployed	Suppliers used	Max employees available	Max trips
Model	19,867,589.33	134,592	31	8	27	1788	325
Independent	34,759,519.33	187,890	31	8	39	1788	323

**Fig. 10:**
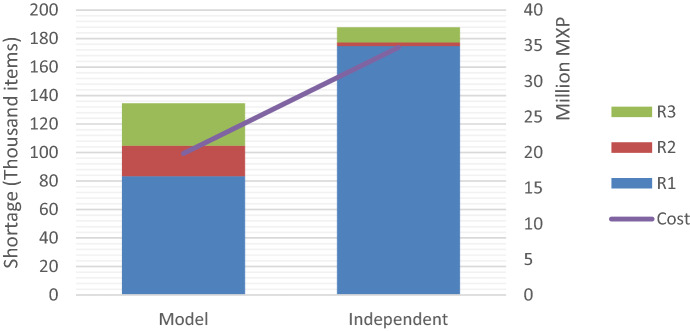
Comparison of the levels of expected shortage per region between the results from the model and planning considering independent disasters

Figure 10 represents shortage across regions and the contrast of cost between both solutions. The three regions were affected by disasters of different magnitude, where region one was the most affected. In the policy proposed by the model, although more than half of the shortage was suffered at region one, the model balances the resources to have levels of 22% of shortage in disaster two, and 23% in disasters one and three. In contrast, independent decision-making worked very well with lower levels of demand, achieving less than 3% and a little over 8% of shortage in regions two and three, respectively. Nevertheless, an expected level of shortage of nearly 48% for region one is troublesome for the most affected area. In summary, the effect of neglecting to consider the occurrence of simultaneous disasters in different areas includes the inability to reach better solutions, the potential to overestimate the resources available and the tendency to increase the number of stakeholders.

Overall, the results show a good level of performance of the model balancing resources for higher and lower levels of demand, trying to use them according to the magnitude, something that is not easily achievable with independent decision-making.

### Sensitivity analysis

**Fig. 11:**
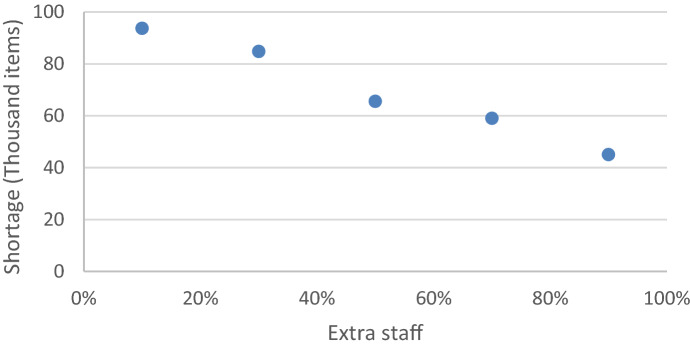
Levels of shortage with extra employees

This article argues that humanitarian logistics formulations need to incorporate human resources because these affect operations performance in practice. To further understand the effect of that component, a sensitivity analysis is included to discuss its impact using the number of employees to show the effect of varying the level of staff on the performance of the system. Figure 11 shows the changes in the value of the minimization of shortage of items when more employees are made available.

The availability of extra staff has an impact in the level of shortage, as evidenced by the potential reduction in more than half of shortage when human resources are increased by 90% compared to the current situation. This result highlights that human resources are one of the critical factors that can affect the delivery of relief to disaster areas, a dimension commonly overlooked in the literature. In simultaneous disasters, the decision about the allocation of staff evolves from establishing who to send, to determine where to send them as well.

## Discussion

### Findings

This article proposes a formulation integrating multiple suppliers, multiple agencies and simultaneous disasters, an area currently understudied. The bi-objective model provides support for decision-making for logistics activities in settings with stochastic demand capable of optimizing the number of agencies deployed to reduce supply congestion in an environment subject to simultaneous disasters.

Planning for simultaneous disasters requires sharing suppliers, resources, and facilities. There is evidence of the benefits of sharing resources among multiple stakeholders (Balcik et al. 2019). The same concept applies for sharing resources among affected regions and crises. The effect that simultaneous disasters have on each other includes the need to share the same pot of resources among different events (Kappes et al. 2012), which effectively means response capabilities for each individual situation are reduced. The relief network needs to be carefully coordinated to allow authorities to stretch their resources to provide assistance in these instances. However, current formulations tend to neglect the inclusion of resources such as vehicles and staff (Rodríguez-Espíndola et al. 2018a). The results from the case study and the sensitivity analysis show the impact human resources can have on disaster operations, especially for developing countries with constrained resources (Julca 2012), such as the case of Mexico.

Supplier selection is a complex endeavor because of the need to balance coverage, supply capacity, delivery times, supply arrangements, and available resources (Aghajani et al. 2020). This is further complicated in simultaneous disasters because the importance of delivery times can promote the use of suppliers closer to one disaster area rather than supplier selection based on a holistic view of the situation across disasters. Additionally, competition for resources (e.g., relief items, vehicles) from different agencies might cause shortages (Balcik and Ak 2014). The results of this research agrees with previous research underscoring the importance of partnerships and agreements with suppliers for disaster response (Wang et al. 2019). Findings suggest that selecting suppliers with more reach and which are better located can be advantageous over multiple localized suppliers for disasters in areas largely separated. Similarly, larger facilities strategically located can be critical to reach different areas and simplify the relief network, leading to lower cost and better service when simultaneous events are considered. This was evident in the comparison between the results of the model and independent response, where it was possible to see the drawbacks of independent decision-making at the planning and response stages.

There is an interesting dynamic captured by the model that would not be evident in current models in the literature. In simultaneous events, resource management becomes a major concern (Doan and Shaw 2019) given varying magnitudes and the need to distribute resources among distinct disaster areas. Opening more facilities and procuring more items requires more investment, and it is more resource intensive as well. Nevertheless, results suggest that vehicles are not a major constraint in the case. The maximum number of trips required by the model was lower than the number of trips that could be done by vehicles available to the agencies deployed. Conversely, the challenge for decision-makers was the availability of human resources. Even when every agency was deployed, there was a shortage of employees for operational activities. On the other hand, the potential of the model to optimize the agencies deployed showed the possibility of reducing the number of participants involved in several solutions in case the decision-maker has more constraints in terms of jurisdiction or operational deployment. The Pareto frontier from the case shows the possibility of obtaining “acceptable” results with less agencies deployed, and the way the number and type of agencies involved should be decided based on their resources, the priorities from decision-makers, and the characteristics of the disasters. Overall, the results of the analysis provide evidence of the potential of the formulation proposed to manage multiple resources from different agencies accounting for the occurrence of simultaneous disasters.

### Practical implications

The formulation proposed can serve as basis to inform disaster managers in countries similar to Mexico. The analysis of the case presented provides a set of implications for managers, such as:

- Planning for simultaneous disasters provides clearer understanding of the real capabilities of the disaster management system and its limitations. It can avoid a false sense of security about resources available coming from the narrow perspective of single independent disasters.
- Efficient operations require accounting for the characteristics, requirements, and priority of the disaster(s) to leverage the interaction of different stakeholders.
- Relief delivery must look at the combination of human and material resources. Neglecting to consider any of them can lead to wasted or insufficient resources.
- Agency deployment requires planning. The deployment of participants must be based on the situation(s) and requirements to avoid overcrowding and duplication of efforts, as human resources have a direct impact on the capabilities of the system and its performance.

## Conclusions and future research

Despite the growing literature in humanitarian logistics, only a handful of articles are looking at the possibility of simultaneous disasters. This paper introduces a novel formulation incorporating the participation of multiple agencies and suppliers for the delivery of relief to disaster victims in multiple regions affected simultaneously, an approach never undertaken before. Although there are formulations emphasizing the importance of multiple suppliers or multiple participants, this is the first formulation integrating multiple suppliers, multiple agencies, and simultaneous disasters in the same model. The application to a case study in Mexico showed the capacity of the model to provide support, tailoring the response based on the scale of the disasters.

The ideas and rationale from this model can be used as the foundation for the development of new holistic decision-support systems integrating different logistics decisions in such a complex environment. Additionally, the results of the case study show the importance of considering suppliers, human resources, and critical facilities to achieve a satisfactory level of service for the victims. Disaster managers can benefit from this research through a well-defined disaster management structure and the impact of supplier selection and management. Aligned with current practices from countries such as the USA, practitioners can use this research to justify the analysis of simultaneous disasters as part of standard practice to have more robust disaster management systems.

Solving the model in a few hours because of the use of discrete variable is feasible at the preparedness stage, but it is desirable to reduce solution times through the development of heuristic algorithms in future work. The model introduced in this article provides support for decisions at the planning stage looking at participant, supplier, and facility selection; thus, it could be coupled with models for logistics decisions to provide integrated support for disaster response for multiple periods. In fact, the development of a dynamic model for multiple disasters to support disaster preparedness and response could help managing subsequent, compound, and simultaneous disasters. Given the uncertainty of simultaneous disasters, new formulations should explore the impact of stochastic supply. This article has shown the importance of looking at human resources for relief operations and the next step should consider incorporating uncertainty in the availability of these resources. The integration of technology with operational research is another interesting stream of research.

## Appendix

### Data description

The model includes information available to the stakeholders involved which is shared with the umbrella organization. These parameters can be clustered in six groups:

- *Procurement data* The type of relief items deployed and their characteristics exist in the list of relief items authorized by authorities and from agreements with suppliers. Procurement costs (prior and after the disaster), partnership cost, minimum order sizes and supply capacity can also be obtained from agreements with contracted suppliers. The procurement budget can be obtained from disaster budgets facilitated by federal, regional or local governments.
- *Facility data* Volumetric capacity of the facilities is information available in the list of pre-selected facilities developed by authorities. Facility costs include all preparations required for the use of the facility (e.g., cleaning, equipment required) and can be obtained from previous reports. Number of employees required per facility is information handled by authorities, usually stating the number of people required to handle a facility of a certain size. From that information, the space covered per employee can be estimated.
- *Agency data* Costs of involving agencies are associated with wages recorded by them and can be obtained from them or through transparency websites. Availability of resources, such as employees and vehicles, can be obtained from their records as well.
- *Transportation data* The cost of transporting relief items can be obtained from direct costs (fuel and vehicle depreciation) using the specification of the vehicles distances. The number of employees required for transportation is information handled by authorities, usually stating the number of people required to make relief distribution trips per vehicle. The capacity of each vehicle can be obtained from the specifications of each type of vehicle.
- *Adjusted parameters* These parameters rely on the guidelines of each decision-maker and their preferences. The coverage of suppliers to facilities depends on time/distance constraints based on the location of the disaster and agreed lead-times. Analysis with GIS can be performed on routes based on the guidelines of the agencies. The number of trips per day relies on the preferred distances of coverage, lead times and working conditions determined by the decision-maker.
- *Scenario-dependent parameters* These parameters rely on the characteristics of the scenarios analyzed, which are decided by authorities based on historical information and forecasting. Relief demand and the probability of the scenarios can be forecasted or obtained from previous reports. Priority of each disaster area per scenario is a parameter that can be adjusted by the decision-maker based on the characteristics and magnitude of the scenario to balance the use of resources among different regions. Simultaneous disasters differ in nature and intensity (Kappes et al. 2012). Hence, this parameter allows the prioritization of the use of resources to provide further support to the most heavily affected regions.

### Case study demand

See Table 13.

**Table 13:** Aggregated demand

Disaster	Scenario	Demand area					
		A1	A2	A3	A4	A5	A6
R1	S1	818,224	46,816	54,131	13,805	169,708	0
R1	S2	818,224	46,816	54,131	13,805	169,708	0
R1	S3	818,224	46,816	54,131	13,805	169,708	0
R1	S4	818,224	46,816	54,131	13,805	169,708	0
R1	S5	818,224	46,816	54,131	13,805	169,708	0
R1	S6	818,224	46,816	54,131	13,805	169,708	0
R1	S7	818,224	46,816	54,131	13,805	169,708	0
R1	S8	818,224	46,816	54,131	13,805	169,708	0
R1	S9	654,577	37,455	43,307	11,044	135,762	0
R1	S10	654,577	37,455	43,307	11,044	135,762	0
R1	S11	654,577	37,455	43,307	11,044	135,762	0
R1	S12	654,577	37,455	43,307	11,044	135,762	0
R1	S13	654,577	37,455	43,307	11,044	135,762	0
R1	S14	654,577	37,455	43,307	11,044	135,762	0
R1	S15	654,577	37,455	43,307	11,044	135,762	0
R1	S16	654,577	37,455	43,307	11,044	135,762	0
R1	S17	0	0	0	0	0	0
R1	S18	0	0	0	0	0	0
R1	S19	0	0	0	0	0	0
R1	S20	0	0	0	0	0	0
R2	S1	161,315	12,595	49,038	102,993	25,971	0
R2	S2	161,315	12,595	49,038	102,993	25,971	0
R2	S3	161,315	12,595	49,038	102,993	25,971	0
R2	S4	129,052	10,076	39,226	82,390	20,779	0
R2	S5	129,052	10,076	39,226	82,390	20,779	0
R2	S6	129,052	10,076	39,226	82,390	20,779	0
R2	S7	0	0	0	0	0	0
R2	S8	0	0	0	0	0	0
R2	S9	161,315	12,595	49,038	102,993	25,971	0
R2	S10	161,315	12,595	49,038	102,993	25,971	0
R2	S11	161,315	12,595	49,038	102,993	25,971	0
R2	S12	129,052	10,076	39,226	82,390	20,779	0
R2	S13	129,052	10,076	39,226	82,390	20,779	0
R2	S14	129,052	10,076	39,226	82,390	20,779	0
R2	S15	0	0	0	0	0	0
R2	S16	0	0	0	0	0	0
R2	S17	161,315	12,595	49,038	102,993	25,971	0
R2	S18	161,315	12,595	49,038	102,993	25,971	0
R2	S19	129,052	10,076	39,226	82,390	20,779	0
R2	S20	129,052	10,076	39,226	82,390	20,779	0
R3	S1	53,372	108,625	37,829	207,119	33,572	63,602
R3	S2	42,691	86,900	30,261	165,693	26,851	50,875
R3	S3	0	0	0	0	0	0
R3	S4	53,372	108,625	37,829	207,119	33,572	63,602
R3	S5	42,691	86,900	30,261	165,693	26,851	50,875
R3	S6	0	0	0	0	0	0
R3	S7	53,372	108,625	37,829	207,119	33,572	63,602
R3	S8	42,691	86,900	30,261	165,693	26,851	50,875
R3	S9	53,372	108,625	37,829	207,119	33,572	63,602
R3	S10	42,691	86,900	30,261	165,693	26,851	50,875
R3	S11	0	0	0	0	0	0
R3	S12	53,372	108,625	37,829	207,119	33,572	63,602
R3	S13	42,691	86,900	30,261	165,693	26,851	50,875
R3	S14	0	0	0	0	0	0
R3	S15	53,372	108,625	37,829	207,119	33,572	63,602
R3	S16	42,691	86,900	30,261	165,693	26,851	50,875
R3	S17	53,372	108,625	37,829	207,119	33,572	63,602
R3	S18	42,691	86,900	30,261	165,693	26,851	50,875
R3	S19	53,372	108,625	37,829	207,119	33,572	63,602
R3	S20	42,691	86,900	30,261	165,693	26,851	50,875
